# The Effect of Organic Acid, Trisodium Phosphate and Essential Oil Component Immersion Treatments on the Microbiology of Cod (*Gadus morhua*) during Chilled Storage

**DOI:** 10.3390/foods7120200

**Published:** 2018-12-08

**Authors:** Conor Smyth, Nigel P. Brunton, Colin Fogarty, Declan J. Bolton

**Affiliations:** 1 Teagasc Food Research Centre, Ashtown, 15 Dublin, Ireland; conor.smyth@teagasc.ie (C.S.); colin.fogarty@teagasc.ie (C.F.); 2 School of Agriculture and Food Science, University College Dublin, Belfield, 4 Dublin, Ireland; nigel.brunton@ucd.ie; 3 School of Veterinary Medicine, University College Dublin, Belfield, 4 Dublin, Ireland

**Keywords:** cod (*Gadus morhua*), microbiology, shelf-life, organic acids, essential oil components

## Abstract

Spoilage is a major issue for the seafood sector with the sale and exportation of fish limited by their short shelf-life. The immediate and storage effects of immersion (30 s at 20 °C) with 5% (*w*/*v*) citric acid (CA), 5% (*v*/*v*) lactic acid (LA), 5% (*w*/*v*) capric acid (CP) and 12% trisodium phosphate (TSP) (experiment 1) and essential oil components (EOC) (1% (*v*/*v*) citral (CIT), 1% (*v*/*v*) carvacrol (CAR), 1% (*w*/*v*) thymol (THY) and 1% (*v*/*v*) eugenol (EUG)) (experiment 2) on the concentrations of indicator (total viable counts (TVC) (mesophilic and psychrophilic) and total *Enterobacteriaceae* counts (TEC)), and spoilage organisms (*Pseudomonas* spp., lactic acid bacteria (LAB), *Brochothrix thermosphacta*, *Photobacterium* spp. and hydrogen sulphide producing bacteria (HSPB)) on cod (*Gadus morhua*) (stored aerobically at 2 °C) was investigated. There was no significant reduction for most treatment-bacteria combinations, with the following exceptions; TSP and TVC_m_ (time *t* = 6), TSP and TVC_p_ (*t* = 6), CP and LAB (*t* = 6, 8 and 10), CP and *Br. thermosphacta* (*t* = 4, 6, 8, 10, 14 and 16), TSP and *Photobacterium* spp. (*t* = 4), CAR and *Br. thermosphacta* (*t* = 6) and CAR and HSPB (*t* = 3, 6, 9, 12, 15 and 18). Although the majority of treatments did not significantly (*P* > 0.05) reduce bacterial counts, the limited success with CP and CAR warrants further investigation.

## 1. Introduction

Gadiformes are a group of fish species, including cod, hake, pollock, whiting, etc., that are commercially important in Europe. Spoilage is a major issue for the seafood sector with approximately 10% of the total catch (12 million tonnes) going to waste every year [1]. Moreover, as the distance between fishing grounds and landing ports gets longer, the scale of losses will increase [2]. Reducing these losses, but still satisfying consumer demand for good quality fish, requires the development of novel preservation technologies. Fish quality begins to deteriorate immediately *post-mortem*. Although enzymatic and chemical reactions are responsible for the initial loss of freshness, most spoilage is due to microbial activity [3]. Thus, fish must be stored at a temperature approaching that of melting ice [4]. However, even at chilled temperatures, psychrotrophic bacteria, mainly psychrotolerant Gram-negative bacteria such as *Pseudomonas* spp. and hydrogen sulphide producing bacteria (HSPB), will grow and spoil the fish [5]. Other bacterial species also associated with fish spoilage include *Brochothrix thermosphacta*, lactic acid bacteria (LAB) and *Enterobacteriaceae* [6,7]. Mesophilic total viable counts (TVC_m_) are therefore widely used as an indicator for shelf-life [8], although psychrophilic total viable count (TVC_p_), total *Enterobacteriaceae* (TEC), *Pseudomonas* spp. and LAB have also been used as quality indicators [9,10].

Preserving the quality of fish such as cod (*Gadus morhua*) is primarily dependent on chilled or frozen storage to retard bacterial growth. Sodium chloride (NaCl) may also be applied but the concentrations required to completely inhibit bacterial growth (9–11% *w*/*w*) adversely affects the sensory attributes of white fish [11,12]. Thus, there is a need for novel fish preservation methods and in recent years there has been increasing interest in the application of chemical preservatives to control microbial, oxidative and autolytic enzymatic spoilage of fish [13]. Of these, organic acids, such as citric acid (CA), lactic acid (LA) and capric acid (CP) are particularly suitable as they occur naturally in foods, are ‘generally recognised as safe’ (GRAS) and are low cost [14]. In addition to lowering the pH of the food, CA and LA molecules penetrate the lipid membrane of bacteria destabilizing the pH in the cytoplasm thereby inhibiting metabolic reactions and growth [15,16]. CP is a medium chain fatty acid that damages the bacterial cell membrane resulting in leakage from the cell [17]. In addition to organic acids, phosphates have also been applied as preservation agents in meat, primarily poultry [18]. Trisodium phosphate (TSP), which reduces the bacterial load by damaging lipid components within the bacterial cell membrane [19,20], has been especially effective [21,22].

There is also a trend towards green consumerism, which has encouraged food processors to find alternatives to synthetic chemicals for food preservation [23]. Essential oils (EO), extracted from plants, herbs and spices, contain naturally occurring antimicrobial compounds and have GRAS status. The antimicrobial properties of EOs are attributed to phenolic compounds such as carvacrol (CAR), thymol (THY) and eugenol (EUG) [24]. Their hydrophobicity allows them to cross the bacterial membrane leading to increased permeability and loss of cellular contents causing cell death [25]. They may also disrupt the proton motive force, electron flow and active transport within the cell and coagulate cell contents [24].

The objective of this study was to investigate the effects of CA, LA, CP, TSP, citral (CIT), CAR, THY and EUG on the growth of indicator (TVC and TEC) and spoilage (*Pseudomonas* spp., LAB, *Br. thermosphacta*, *Photobacterium* spp. and HSPB) bacteria on cod stored aerobically at 2 °C.

## 2. Materials and Methods

### 2.1. Fish Supply

Whole fresh cod were obtained from a local fishmonger, transported to Teagasc Food Research Centre (Ashtown, Dublin, Ireland) in an insulated cooler box within 1 h of purchase and stored whole on ice in a polystyrene box in a chill room at 2 °C. The fish were obtained within 48 h of landing and were of a similar weight (2.5–3 kg).

### 2.2. Sample Preparation and Storage

The cod were aseptically filleted and cut into portions weighing approximately 10 g each, of similar dimensions and surface area. Samples were treated using a 30 s dip (20 °C) in the following solutions; 5% (*w*/*v*) CA (Sigma Aldrich, Steinheim, Germany), 5% (*v*/*v*) LA (Sigma Aldrich, Steinheim, Germany), 5% (*w*/*v*) CP (Sigma Aldrich, Steinheim, Germany), 12% (*w*/*v*) TSP (Sigma Aldrich, Steinheim, Germany), 1% (*v*/*v*) CIT (Sigma Aldrich, Steinheim, Germany), 1% (*v*/*v*) CAR (Sigma Aldrich, Steinheim, Germany), 1% (*v*/*v*) THY (Sigma Aldrich, Steinheim, Germany) and 1% (*v*/*v*) EUG (Sigma Aldrich, Steinheim, Germany). Untreated samples and samples dipped in sterile distilled water (SDW) were used as controls. After each immersion, the samples were removed and the excess allowed to drip off for 15 s. The samples were then immersed in SDW for 30 s as a rinse step, to remove excess treatment residue and again given 15 s to drip off. Samples were then stored aerobically at 2 °C until required.

### 2.3. Microbiological Analysis

In experiment 1, microbiological analysis was performed on the 5% organic acid and 12% TSP treated samples at times (*t*) = 0, 2, 4, 6, 8, 10, 12, 14, 16 and 18 days. In experiment 2, the lower concentration treatments (1% essential oil components (EOC)) were sampled at times (*t*) = 0, 3, 6, 9, 12, 15 and 18 days. At each sampling time, the fish samples (10 g) were placed in filtered stomacher bags with 90 mL maximum recovery diluent (MRD) (Oxoid, Basingstoke, UK (CM0733)) and homogenised in a stomacher (Pulsifier^®^ PUL100E, Microgen Bioproducts Ltd., Surrey, UK) for 60 s and a 10-fold dilution series prepared using MRD. TVC_m_ and TVC_p_ were determined using plate count agar (PCA, Oxoid, Basingstoke, UK (CM0325)) incubated at 30 °C for 72 h and 6.5 °C for 10 days, respectively. *Enterobacteriaceae* counts were carried out using violet red bile glucose agar (VRBGA) (Oxoid, Basingstoke, UK (CM0485)) incubated at 37 °C for 24 h. *Pseudomonas* spp. were determined using *Pseudomonas* agar base (Oxoid, Basingstoke, UK (CM0559)) with Cephalothin-Sodium Fusidate-Cetrimide (CFC) supplement (Oxoid, Basingstoke, UK (SR0103)) incubated at 30 °C for 48 h. LAB were cultured using de Man Rogosa Sharpe (MRS) agar (Oxoid, Basingstoke, UK (CM0361)) incubated at 30 °C for 72 h. *Br. thermosphacta* was tested using Streptomycin-thallous acetate-actidione (STAA) agar base (Oxoid, Basingstoke, UK (CM0881)) with STAA selective supplement (Oxoid, Basingstoke, UK (SR0151E)) incubated at 25 °C for 72 h. *Photobacterium* spp. were enumerated on *Photobacterium* broth (Sigma Aldrich, Steinheim, Germany (38719-500G-F)) with bacteriological No.1 agar (Oxoid, Basingstoke, UK (LP0011)) added as per the instructions and incubated at 15 °C for 7 days. HSPB were enumerated on Iron Lyngby (IL) agar as described by the Nordic Committee on Food Analysis (NMKL) (method 184, 2006) [26], supplemented with L-cysteine (Sigma Aldrich, Steinheim, Germany) and incubated at 25 °C for 72 h.

### 2.4. Physical Measurements

The pH of samples was measured using a Eutech Instruments pH 5+ pH meter (Thermo Fisher Scientific, Dublin, Ireland). The water activity (a_w_) of samples was recorded using a Decagon AquaLab LITE water activity meter (Labcell Ltd., Alton, UK) as per the manufacturer’s instructions. The thickness, length and width of samples were recorded to determine the average surface area, with log values of microbial counts being expressed as CFU/cm^2^.

### 2.5. Statistical Analysis

All experiments were undertaken in duplicate and repeated on 3 separate occasions. Bacterial counts were converted to log_10_ CFU/cm^2^. Mean generation times (*G*) for bacteria (from time *t* = 0 to the time where the highest bacterial concentration was recorded) were calculated using the formula:

*G* = *t*/3.3·log*b*/*B* (1)

where *t* = time interval in h, *b* = number of bacteria at the end of the time interval, and *B* = number of bacteria at the beginning of the time interval [27].

The difference between mean values was compared using a two way analysis of variance (ANOVA). GraphPad Prism v7.0 (GraphPad Software Inc., La Jolla, CA, USA) was used for statistical analysis, and significant differences reported at *P* < 0.05.

## 3. Results

The mean pH and a_w_ for cod treated with 5% CA, LA, CP or 12% TSP (experiment 1) and stored for 18 days at 2 °C are shown in Table 1. The initial pH value for the untreated control was 6.4, which increased to 8.5 by day 16. The pH values for the treated samples ranged between 5.4 and 8.8 on day 0 and 8.0 to 8.7 on day 18. The a_w_ for the untreated control and treated samples ranged between 0.93 and 0.99 throughout the 18 day storage period. There was no significant (*P* > 0.05) difference in the pH or a_w_ values for CA, LA or CP treated samples throughout the 18 days of storage. Indeed, the only significant difference was observed in the pH of TSP treated samples at time *t* = 0 days.

The pH and a_w_ values for samples treated with 1% CIT, CAR, THY, or EUG (experiment 2) stored for 18 days at 2 °C are shown in Table 2. The initial pH (*t* = 0) for the untreated control was 7.0, increasing to 8.5 after 18 days storage. The pH of treated samples ranged between 6.8 and 6.9 on day 0 and 8.3 to 8.5 on day 18. The a_w_ ranged between 0.99 and 1.00 throughout storage, regardless of the treatment type. There was no significant difference between the untreated control and treated samples throughout experiment 2. The SEM for the pH values ranged between 0 and 0.3 while the highest SEM for the a_w_ values was 0.01.

The mean bacterial (TVC_m_, TVC_p_, TEC, *Pseudomonas* spp., LAB, *Br. thermosphacta*, *Photobacterium* spp. and HSPB) counts on cod treated with 5% (*w*/*v*) CA, 5% (*v*/*v*) LA, 5% (*w*/*v*) CP and 12% (*w*/*v*) TSP and stored at 2 °C for 18 days are shown in Table 3. The corresponding growth parameters (initial lag period, mean generation time and maximum growth rate) are shown in Table 4.

TVC_m_ and TVC_p_ counts were statistically similar (*P* > 0.05) to the control at each sampling time, regardless of treatment, with the exception of TVC_m_ with TSP and TVC_p_ with TSP, both at *t* = 6. A similar trend was observed with the counts of other bacterial groups (TEC, *Pseudomonas* spp., LAB, *Br. thermosphacta, Photobacterium* spp. and HSPB) with the following exceptions; LAB with CP (*t* = 6, 8 and 10), *Br. thermosphacta* with CP (*t* = 4, 6, 8, 10, 14 and 16) and *Photobacterium* spp. with TSP (*t* = 4), all of which were significantly (*P* > 0.05) lower than the untreated control. An initial lag period, before the bacterial cultures entered the logarithmic phase of growth, was only observed with the following combinations; CTL (LAB), CA (TEC, LAB and *Photobacterium* spp.), LA (TEC and LAB), CP (TEC, LAB, *Br. thermosphacta* and *Photobacterium* spp.) and TSP (LAB, *Br. thermosphacta* and *Photobacterium* spp.). There was no pattern to these observations with the initial lag period ranging from 15.6 h (untreated control-LAB) to 103.7 h (CP-LAB). The mean generation times for treated samples were higher than both the untreated control and SDW treated samples with the following: CA (TVC_m_, TVC_p_, *Br. thermosphacta*, *Photobacterium* spp. and HSPB), LA (TVC_p_, *Pseudomonas* spp., *Br. thermosphacta* and HSPB), CP (TVC_m_, *Pseudomonas* spp., LAB, *Br. thermosphacta*, and HSPB) and TSP (TVC_m_, TVC_p_ and *Pseudomonas* spp.). The maximum growth rates ranged from 0.02 to 0.08 generations h^−1^, regardless of treatment.

An initial lag period was observed with the majority of the samples. This ranged from 3.2 to 127.3 h and, as in experiment 1, there was no pattern (i.e., a consistently longer lag period for a given treatment as compared to the controls was not observed). Moreover, the mean generation times for treated samples were similar or lower than the untreated and/or the SDW treated controls with the exception of THY-TEC and THY-LAB which at 42.4 h and 27.5 h were approximately 3 h and 1 h longer than the controls. The maximum growth rates ranged from 0.02 to 0.1 generations h^−1^, regardless of treatment.

## 4. Discussion

The initial TVC levels were similar (3–4 log_10_ CFU/cm^2^) to those that have been previously reported on fresh cod [6,28], fresh herring [29,30,31] and fresh carp [32]. Interestingly, in experiment 1, the TVC_p_ growth rate (mean generation time of 15.6 h) was higher than that observed with the TVC_m_ (18.9 h), as previously reported on herring stored at 4 °C [31]. The overall increase in TVC_m_ and TVC_p_ (to 8.0 and 8.4 log_10_ CFU/cm^2^, respectively) were similar to those previously reported for cod (3.2 log_10_ CFU/cm^2^ after 12 days at 0 °C) [6] and carp (4 log_10_ CFU/cm^2^ after 14 days at 4 °C) [32]. The initial TEC suggested that our cod was caught in unpolluted waters, processed hygienically and chilled quickly [33,34,35]. The TEC obtained throughout 18 days of 3.6 and 2.8 log_10_ CFU/cm^2^ for experiments 1 and 2 respectively, were also below the acceptable limit of 4 log_10_ CFU/cm^2^ for white fish [30].

There was an overall lack of inhibition by CA, LA, CP and TSP on TVC_m_, TVC_p_ and TEC, which is in contrast to previous work. García-Soto et al. [36] obtained TVC_m_ and TVC_p_ reductions of up to 2.5 and 1.5 log_10_ CFU/cm^2^, respectively, on hake (*Merluccius merluccius*) stored in ice that included 0.175/0.050% CA/LA and Sallam [37] observed a 5 to 8 day increase in the shelf-life of refrigerated (1 °C) salmon (*Onchorhynchus nerka*) treated with sodium citrate, sodium lactate and sodium acetate. The difference in efficacy of the organic acid treatments on fish may be a result of the different application methods and the impact of the pH, leading to the organic acids being in their dissociated form. In our study, the fish were treated by immersing in the solution for 30 s followed by a rinse step in SDW to remove excess acid. Sallam [37] also used a dip method for 10 min, with no rinse step, while García-Soto et al. [36] applied an ice slurry containing the organic acids. The pH of the fish in our study was reduced from 6.4 to 5.4; however, Dibner and Buttin [16], state that many organic acids must have a pH of between 3 and 5 to be in their undissociated form, thus allowing them to diffuse across the bacterial membrane and disrupt the cell metabolism [17]. Moreover, the possibility that the high protein content of the cod may have acted as a buffer, negating the effectiveness of the organic acids cannot be excluded [38].

The varying success of the treatments raise important issues about treatment application as there is no standard procedure. Currently, European authorities do not permit chemical decontamination in fresh fish; however, the European Food Safety Authority (EFSA) has indicated that any consideration of these decontamination methods in the future would require the experiments to include a rinse step [39].

TSP has been shown to be effective in the decontamination of poultry [40]; however, to the best of our knowledge there are no studies reporting the treatment of fresh fish. The apparent inability of 12% TSP to remove and/or inhibit bacterial growth in our study may be due to the rinse step after treatment. Although the primary mechanism of TSP is physical detachment of cells [41], lysis of bacterial cells may occur during subsequent storage but only if the residual TSP has not been removed by rinsing in water [19].

As with the organic acids, there was very limited bacterial inhibition after treatment with CIT, CAR, THY and EUG on TVC_m_, TVC_p_ and TEC. An initial reduction was achieved with each of the EOCs; however, this effect was lost by day 6. This was in agreement with Van Haute et al. [42], who found that there was little effect on salmon after treatment with 1% oregano and 1% thyme oil after 6 days storage. However, reductions of 1 and 2 log_10_ CFU/cm^2^ have also been reported with sea bass after treatment with oregano and thyme oil, increasing shelf-life from 12 to 33 days [43], while CAR and THY have been shown to reduce TVC levels in carp both individually and by up to 3 log_10_ CFU/cm^2^ when combined in a 1% mixture [23].

As with the organic acids, the limited success of EOC may be a result of the pH of the treated fish (6.8–6.9), as a lower pH increases bacterial susceptibility by increasing the hydrophobicity allowing the essential oil to dissolve the cell membrane [24]. The low storage temperature [44], atmospheric oxygen levels [24] and use of individual rather than mixed (which have a synergistic effect) treatments may have affected the inhibition of bacteria [45,46].

In general, CA, LA, CP, TSP, CIT, CAR, THY and EUG treatments did not significantly (*P* > 0.05) affect the *Pseudomonas* spp., LAB, *Br. thermosphacta*, *Photobacterium* spp. or HSPB counts on cod fillets. However, LAB and *Br. thermosphacta* counts, were significantly (*P* < 0.05) lower on CP treated samples until day 10 of the experiment. CP is a saturated medium chain fatty acid that may inhibit Gram positive bacteria, such as LAB and *Br. thermosphacta*, by disrupting the cell membrane causing leakage from the cell [17,47]. CP inhibited initial growth, extending the initial lag time of LAB from 15.6 h (untreated control) to 104 h and stopping *Br. thermosphacta* (which was already in the early log phase of growth at time *t* = 0) for 83 h. The corresponding mean generation times (36.6 h and 96.6 h) were also increased as compared to the untreated samples (27.8 and 16.6 h). CAR is a phenolic compound that may also disrupt bacterial membrane function [48,49] and in our study significantly reduced HSPB throughout storage, possibly because growth was stopped and the cells entered an extended lag period (78 h). In similar studies Mexis et al. [50] observed a reduction of 1.5 log_10_ CFU/g in HSPB on fish after treatment with oregano oil, of which CAR is a major antimicrobial component as did Teixeira et al. [51] who observed that both *Br. thermosphacta* and *Shewanella putrefaciens* (a prominent HSPB) were sensitive to treatment with thyme oil.

## 5. Conclusions

Overall, the treatments of CA, LA, CP, TSP, CIT, CAR, THY and EUG were not successful in inhibiting growth and extending the shelf-life of cod. This may be attributed to the relatively low concentrations used, the organic acids being in the dissociated form and/or the buffering capacity of the cod proteins. The availability of nutrients in the fish to support bacterial repair and growth may also have been a contributory factor. However, the limited success of various antimicrobials, mainly CP and CAR warrants further investigation.

## Figures and Tables

**Table 1:** pH and water activity (a_w_) measurements (and standard error of the mean (SEM)) for cod treated with 5% (*w*/*v*) citric acid (CA), 5% (*v*/*v*) lactic acid (LA), 5% (*w*/*v*) capric acid (CP) or 12% (*w*/*v*) trisodium phosphate (TSP) and stored at 2 °C for 18 days.

Time (days)	Treatment											
	CTL	SEM	SDW	SEM	CA	SEM	LA	SEM	CP	SEM	TSP	SEM
**pH**												
0	6.4 ^A,1^	0.7	6.3 ^A^	0.6	5.4 ^A^	0.4	5.8 ^A^	0.6	6.4 ^A^	0.4	8.8 ^B^	0.9
2	6.4	0.4	6.6	0.4	6.3	0.3	6.6	0.4	6.7	0.3	7.7	0.3
4	7.1 ^A,B^	0.2	6.9 ^A,B^	0.0	6.7 ^A,B^	0.1	6.3^A^	0.2	6.9 ^A,B^	0.1	7.9 ^B^	0.6
6	7.2	0.9	7.3	0.6	6.8	0.5	6.9	0.3	7.1	0.3	8.0	0.3
8	7.9	0.8	7.5	0.4	7.3	0.2	7.7	0.5	7.4	0.0	8.4	0.4
10	8.1	0.4	8.0	0.2	7.2	0.5	7.2	0.4	7.5	0.2	8.4	0.4
12	8.2	0.9	7.9	1.1	8.1	0.9	8.1	0.6	8.1	0.5	8.4	0.7
14	8.4	0.3	8.0	0.6	7.8	0.5	7.7	0.4	7.8	0.4	8.4	0.3
16	8.5	0.5	8.3	0.7	8.2	0.4	8.1	0.3	8.0	0.4	8.8	0.4
18	8.2	0.3	8.4	0.4	8.4	0.4	8.3	0.3	8.0	0.3	8.7	0.2
**a_w_**												
0	0.98	0.01	0.98	0.01	0.98	0.01	0.98	0.01	0.98	0.01	0.97	0.01
2	0.97	0.01	0.97	0.01	0.97	0.01	0.96	0.01	0.97	0.01	0.96	0.01
4	0.96	0.01	0.97	0.01	0.97	0.01	0.97	0.01	0.97	0.01	0.96	0.01
6	0.96	0.01	0.97	0.01	0.97	0.00	0.97	0.01	0.97	0.01	0.96	0.00
8	0.96	0.01	0.97	0.00	0.97	0.00	0.97	0.00	0.97	0.01	0.96	0.00
10	0.98	0.00	0.98	0.00	0.98	0.01	0.98	0.00	0.98	0.00	0.98	0.00
12	0.93	0.00	0.94	0.00	0.95	0.00	0.95	0.00	0.95	0.00	0.95	0.00
14	0.95	0.01	0.97	0.00	0.96	0.01	0.98	0.00	0.97	0.01	0.96	0.01
16	0.97	0.01	0.95	0.03	0.95	0.03	0.97	0.03	0.95	0.02	0.96	0.01
18	0.98	0.00	0.99	0.00	0.99	0.00	0.99	0.00	0.98	0.02	0.96	0.00

^1^ Statistical analysis: At a given sampling time the same letter indicates no significant (*P* > 0.05) difference. Absence of a letter also indicates no significant (*P* > 0.05) difference.

**Table 2:** pH and water activity (a_w_) measurements (and standard error of the mean (SEM)) for cod treated with 1% (*v*/*v*) citral (CIT), 1% (*v*/*v*) carvacrol (CAR), 1% (*w*/*v*) thymol (THY) or 1% (*v*/*v*) eugenol (EUG) and stored at 2 °C for 18 days.

Time (days)	Treatment											
	CTL	SEM	SDW	SEM	CIT	SEM	CAR	SEM	THY	SEM	EUG	SEM
**pH**												
0	7.0	0.0	6.8	0.0	6.8	0.1	6.9	0.1	6.9	0.1	6.9	0.1
3	7.0	0.1	7.0	0.1	7.0	0.2	6.9	0.0	7.0	0.1	6.9	0.1
6	7.1	0.1	7.0	0.1	7.1	0.2	7.0	0.1	7.0	0.1	7.0	0.1
9	7.2	0.2	7.2	0.2	7.5	0.2	7.1	0.2	7.3	0.3	7.2	0.2
12	8.1	0.2	8.1	0.3	7.8	0.2	7.7	0.3	7.9	0.3	7.9	0.3
15	8.2	0.1	8.2	0.0	8.2	0.1	8.0	0.2	8.2	0.2	8.3	0.2
18	8.5	0.1	8.4	0.2	8.5	0.2	8.3	0.2	8.5	0.2	8.4	0.3
**a_w_**												
0	0.99	0.00	0.99	0.00	0.99	0.00	0.99	0.01	1.00	0.01	1.00	0.01
3	0.99	0.00	0.99	0.00	0.99	0.00	0.99	0.00	0.99	0.00	0.99	0.00
6	0.99	0.00	0.99	0.00	0.99	0.00	0.99	0.00	0.99	0.00	0.99	0.00
9	0.99	0.00	0.99	0.00	0.99	0.00	0.99	0.00	0.99	0.00	0.99	0.00
12	0.99	0.00	0.99	0.00	0.99	0.00	0.99	0.00	0.99	0.00	0.99	0.00
15	0.99	0.00	0.99	0.00	0.99	0.00	0.99	0.00	0.99	0.00	0.99	0.00
18	0.99	0.00	0.99	0.00	0.99	0.00	0.99	0.00	0.99	0.00	0.99	0.00

^1^ Statistical analysis: At a given sampling time the same letter indicates no significant (*P* > 0.05) difference. Absence of a letter also indicates no significant (*P* > 0.05) difference.

**Table 3:** Mean mesophilic total viable count (TVC_m_), psychrophilic total viable count (TVC_p_), total *Enterobacteriaceae* (TEC), *Pseudomonas* spp., lactic acid bacteria (LAB), *Br. thermosphacta*, *Photobacterium* spp. and hydrogen sulphide producing bacteria (HSPB) counts (log_10_ CFU/cm^2^) on cod treated with sterile distilled water (SDW), 5% (*w*/*v*) citric acid (CA), 5% (*v*/*v*) lactic acid (LA), 5% (*w*/*v*) capric acid (CP) or 12% (*w*/*v*) trisodium phosphate (TSP) and stored at 2 °C for 18 days.

Time (days)	Treatment											
	CTL ^1^		SDW		CA		LA		CP		TSP	
**TVC_m_**												
	Log	SE	Log	SE	Log	SE	Log	SE	Log	SE	Log	SE
**0**	3.9	0.2	3.7	0.1	3.7	0.1	3.3	0.4	3.6	0.1	3.4	0.2
**2**	4.8	0.2	4.9	0.3	4.6	0.4	4.1	0.4	5.2	0.4	4.0	0.4
**4**	6.1 ^B,2^	0.2	6.3 ^B^	0.3	5.9 ^B^	0.7	5.4 ^A,B^	0.4	5.6 ^A,B^	0.7	4.6 ^A^	0.2
**6**	6.7 ^A,B^	0.4	7.2 ^B^	0.2	6.1 ^A,B^	0.4	6.7 ^A,B^	0.1	6.4 ^A,B^	0.5	5.7 ^A^	0.2
**8**	7.1	0.1	7.1	0.2	6.7	0.7	6.8	0.2	7.0	0.3	6.4	0.5
**10**	7.6	0.3	7.9	0.1	7.8	0.5	7.8	0.1	7.7	0.4	6.9	0.3
**12**	7.9	0.3	7.1	0.4	7.9	0.1	7.8	0.4	8.0	0.3	7.4	0.4
**14**	7.9	0.2	7.4	0.3	7.6	0.1	8.2	0.5	7.8	0.5	7.1	0.5
**16**	8.0	0.1	8.3	0.2	9.0	0.6	8.5	0.5	8.1	0.2	8.2	0.3
**18**	7.9	0.1	7.9	0.4	8.3	0.3	8.7	0.3	8.3	0.2	8.0	0.4
**TVC_p_**												
	Log	SE	Log	SE	Log	SE	Log	SE	Log	SE	Log	SE
**0**	3.7	0.2	4.0	0.4	3.3	0.3	3.0	0.5	3.2	0.3	2.9	0.2
**2**	4.6	0.5	5.0	0.5	4.5	0.9	4.3	0.2	4.5	0.8	4.2	0.4
**4**	6.2 ^B^	0.3	6.5 ^B^	0.3	5.3 ^A,B^	0.9	5.4 ^A,B^	0.2	5.3 ^A,B^	0.8	4.5 ^A^	0.6
**6**	6.8	0.4	7.2	0.2	6.3	0.8	6.7	0.3	6.3	0.9	5.8	0.6
**8**	7.6	0.3	7.6	0.2	7.2	0.4	7.1	0.2	7.4	0.4	6.7	0.5
**10**	7.7	0.4	8.0	0.2	7.9	0.4	7.8	0.4	7.9	0.5	7.1	0.4
**12**	8.3	0.2	7.7	0.6	8.0	0.3	7.8	0.4	8.6	0.1	7.6	0.2
**14**	8.4	0.1	7.8	0.4	8.1	0.1	8.3	0.4	8.2	0.1	7.8	0.3
**16**	8.2	0.0	8.4	0.2	8.9	0.6	8.5	0.2	8.3	0.1	8.2	0.2
**18**	8.1	0.2	8.1	0.3	8.5	0.2	9.2	0.3	8.6	0.2	8.2	0.1
**TEC**												
	Log	SE	Log	SE	Log	SE	Log	SE	Log	SE	Log	SE
**0**	1.4	0.7	1.5	0.6	1.2	0.4	1.3	0.6	1.4	0.4	0.4	0.4
**2**	1.2	0.7	1.6	0.4	1.3	0.5	1.5	0.4	1.3	0.6	1.1	0.1
**4**	2.2	0.4	2.5	0.3	1.9	0.4	1.9	0.3	2.0	0.5	1.6	0.5
**6**	2.3	0.6	2.6	0.2	1.5	0.5	2.1	0.7	2.2	0.5	1.6	0.8
**8**	2.5	0.7	2.6	0.3	2.2	0.7	2.7	0.7	2.4	0.6	2.1	0.8
**10**	3.0	0.8	3.3	0.4	2.7	0.8	3.3	1.2	2.6	0.6	2.9	0.8
**12**	2.5	0.5	3.4	0.1	3.1	0.4	2.9	1.2	3.0	0.3	2.7	0.9
**14**	3.5	0.6	3.4	0.5	3.4	0.7	3.5	0.8	3.6	0.4	2.9	0.7
**16**	3.6	0.6	3.6	0.4	3.5	0.7	4.1	0.7	3.3	0.4	3.5	1.0
**18**	3.5	0.6	4.0	0.6	3.6	0.8	4.4	0.7	4.2	0.5	3.8	0.6
***Pseudomonas* spp.**												
	Log	SE	Log	SE	Log	SE	Log	SE	Log	SE	Log	SE
**0**	3.5	0.5	3.6	0.5	3.1	0.5	2.6	0.6	3.3	0.6	2.5	0.5
**2**	4.8	0.4	5.1	0.2	4.3	0.7	4.5	0.3	4.4	0.6	3.7	0.6
**4**	6.2 ^A,B^	0.3	6.5 ^B^	0.3	5.8 ^A,B^	0.6	5.3 ^A,B^	0.3	6.0 ^A,B^	0.4	4.7 ^A^	0.2
**6**	7.0	0.4	7.3	0.2	6.3	0.6	6.9	0.1	6.4	0.7	5.7	0.3
**8**	7.7	0.3	7.6	0.3	7.5	0.6	7.2	0.3	7.6	0.3	6.8	0.4
**10**	7.8	0.4	8.5	0.3	8.3	0.4	8.0	0.3	8.1	0.3	7.4	0.5
**12**	8.1	0.3	8.0	0.4	8.4	0.1	8.2	0.2	8.9	0.2	7.8	0.3
**14**	8.7	0.3	8.1	0.5	7.9	0.4	8.6	0.4	8.8	0.4	7.9	0.6
**16**	8.3	0.2	8.8	0.3	9.2	0.9	8.7	0.2	8.6	0.4	8.3	0.3
**18**	7.8 ^A^	0.3	8.2 ^A,B^	0.4	8.8 ^A,B^	0.3	9.5 ^B^	0.6	9.0 ^A,B^	0.4	8.7 ^A,B^	0.5
**LAB**												
	Log	SE	Log	SE	Log	SE	Log	SE	Log	SE	Log	SE
**0**	2.6	0.6	2.6	0.5	2.5	0.4	2.0	0.4	2.4	0.4	2.5	0.5
**2**	2.8	0.5	3.0	0.2	2.6	0.4	2.3	0.4	2.3	0.3	2.5	0.4
**4**	3.4 ^A,B^	0.4	3.8 ^B^	0.1	3.1 ^A,B^	0.4	2.7 ^A,B^	0.4	2.3 ^A^	0.4	3.0 ^A,B^	0.4
**6**	4.1 ^B^	0.5	4.5 ^B^	0.0	3.3 ^A,B^	0.5	3.8 ^A,B^	0.5	2.8 ^A^	0.4	4.0 ^A,B^	0.5
**8**	4.5 ^B^	0.6	4.6 ^B^	0.2	4.1 ^A,B^	0.5	4.5 ^B^	0.4	3.2 ^A^	0.3	5.1 ^B^	0.6
**10**	4.7 ^B^	0.5	5.1 ^B^	0.3	4.6 ^B^	0.5	5.0 ^B^	0.6	3.2 ^A^	0.4	5.5 ^B^	0.5
**12**	5.4 ^A,B^	0.7	5.5 ^A,B^	0.8	5.4 ^A,B^	0.3	5.4 ^A,B^	1.0	4.4 ^A^	0.4	6.2 ^B^	0.4
**14**	5.4 ^A,B^	0.4	5.3 ^A,B^	0.5	5.0 ^A,B^	0.6	5.5 ^A,B^	0.7	4.2 ^A^	0.8	6.0 ^B^	0.2
**16**	5.5 ^A,B^	0.3	5.6 ^B^	0.3	5.7 ^B^	0.6	6.0 ^B^	0.3	4.3 ^A^	0.9	6.1 ^B^	0.3
**18**	5.7 ^A,B^	0.4	5.9 ^A,B^	0.4	6.0 ^A,B^	0.3	6.3 ^A,B^	0.2	5.0 ^A^	1.0	6.5 ^B^	0.1
***Br. Thermosphacta***												
	Log	SE	Log	SE	Log	SE	Log	SE	Log	SE	Log	SE
**0**	2.3	0.4	2.3	0.3	1.9	0.4	1.7	0.5	1.5	0.4	1.5	0.4
**2**	3.3	0.5	3.4	0.2	2.8	0.7	2.8	0.2	2.1	0.6	2.4	0.7
**4**	4.5 ^B^	0.4	5.1 ^B^	0.1	3.8 ^B^	0.8	3.5 ^A,B^	0.4	2.3 ^A^	0.4	3.2 ^A,B^	0.5
**6**	5.3 ^B^	0.5	5.5 ^B^	0.2	4.6 ^B^	0.8	5.0 ^B^	0.4	2.8 ^A^	0.5	4.5 ^B^	0.7
**8**	6.2 ^B^	0.5	6.1 ^B^	0.3	5.3 ^A,B^	0.9	5.3 ^A,B^	0.4	3.8 ^A^	0.5	5.5 ^B^	0.5
**10**	6.2 ^B^	0.4	6.6 ^B^	0.4	6.2 ^B^	0.5	6.2 ^B^	0.3	4.9 ^A^	0.1	6.2 ^B^	0.6
**12**	6.7	0.6	6.6	0.6	6.5	0.2	6.2	0.6	5.4	0.0	6.5	0.4
**14**	6.8 ^B^	0.2	6.6 ^A,B^	0.3	6.3 ^A,B^	0.5	6.5 ^A,B^	0.4	5.4 ^A^	0.2	6.7 ^A,B^	0.2
**16**	6.9 ^B^	0.2	7.2 ^B^	0.3	7.0 ^B^	0.6	7.0 ^B^	0.2	5.2 ^A^	0.4	6.9 ^B^	0.4
**18**	7.0	0.2	7.0	0.3	7.0	0.3	7.2	0.2	5.9	0.4	7.3	0.3
***Photobacterium* spp.**												
	Log	SE	Log	SE	Log	SE	Log	SE	Log	SE	Log	SE
**0**	4.1	0.0	3.9	0.1	3.9	0.1	3.3	0.7	3.8	0.0	3.7	0.0
**2**	4.5	0.2	4.4	0.1	4.3	0.4	4.2	0.2	4.2	0.3	3.7	0.2
**4**	5.8 ^B^	0.1	5.6 ^A,B^	0.4	4.8 ^A,B^	0.5	5.5 ^A,B^	0.3	4.9 ^A,B^	0.2	4.1 ^A^	0.3
**6**	6.8	0.6	6.6	0.2	6.1	1.1	6.7	0.4	6.1	1.0	5.4	0.6
**8**	7.3	0.3	7.2	0.3	6.7	0.8	7.3	0.2	7.2	0.4	6.8	0.7
**10**	7.3	0.4	7.3	0.3	7.0	0.8	7.7	0.2	7.1	0.7	6.5	0.5
**12**	8.0	n/a	8.5	n/a	8.2	n/a	8.5	n/a	8.6	n/a	8.0	n/a
**14**	8.2	0.6	8.0	0.2	7.7	0.6	8.2	0.2	8.0	0.5	7.7	0.7
**16**	7.9	0.2	8.0	0.1	7.9	0.5	8.3	0.3	8.1	0.3	8.3	0.1
**18**	8.2	0.2	8.2	0.1	8.9	0.1	9.2	0.4	9.0	0.3	8.9	0.5
**HSPB**												
	Log	SE	Log	SE	Log	SE	Log	SE	Log	SE	Log	SE
**0**	3.0	0.5	3.1	0.4	2.5	0.5	2.4	0.8	2.0	0.5	2.1	0.2
**2**	4.4	0.5	4.7	0.3	3.3	1.0	3.6	0.2	3.6	0.9	3.6	0.8
**4**	5.4 ^A,B^	0.1	5.9 ^B^	0.2	4.1 ^A^	0.8	4.5 ^A,B^	0.3	4.4 ^A,B^	0.4	4.4 ^A,B^	0.4
**6**	6.4 ^A,B^	0.5	7.1 ^B^	0.2	5.0 ^A^	0.9	5.7 ^A,B^	0.3	5.5 ^A,B^	0.9	5.7 ^A,B^	0.6
**8**	7.1	0.3	7.1	0.2	5.6	0.8	5.9	0.4	6.2	0.4	6.9	0.4
**10**	7.2	0.2	7.3	0.4	6.2	1.0	6.0	0.5	6.7	0.6	7.1	0.5
**12**	7.4	0.3	7.5	0.3	6.8	0.1	6.5	0.2	7.0	0.5	7.6	0.2
**14**	7.5	0.1	7.4	0.2	6.4	0.3	6.7	0.2	6.9	0.4	7.2	0.2
**16**	7.6	0.1	7.9	0.3	6.9	0.6	6.9	0.2	6.8	0.5	7.5	0.2
**18**	7.6	0.1	7.3	0.1	7.0	0.3	7.2	0.3	7.1	0.4	7.6	0.2

^1^ CTL = untreated control; ^2^ Statistical analysis: At a given sampling time the same letter indicates no significant (*P* > 0.05) difference. Absence of a letter also indicates no significant (*P* > 0.05) difference.

**Table 4:** Growth parameters (initial lag period, mean generation time and maximum growth rate (µmax)) of mesophilic total viable count (TVC_m_), psychrophilic total viable count (TVC_p_), total *Enterobacteriaceae* (TEC), *Pseudomonas* spp., lactic acid bacteria (LAB), *Br. thermosphacta*, *Photobacterium* spp. and hydrogen sulphide producing bacteria (HSPB) on cod treated with sterile distilled water (SDW), 5% (*w*/*v*) citric acid (CA), 5% (*v*/*v*) lactic acid (LA), 5% (*w*/*v*) capric acid (CP) or 12% (*w*/*v*) trisodium phosphate (TSP) and stored aerobically at 2 °C for 18 days.

Bacteria	Initial Lag Time (h)						Mean Generation Time (h) ^1^						µmax (Generations h^−1^)					
	CTL ^2^	SDW	CA	LA	CP	TSP	CTL	SDW	CA	LA	CP	TSP	CTL	SDW	CA	LA	CP	TSP
**TVC_m_**	NA ^3^	NA	NA	NA	NA	NA	18.9	12.8	23.3	18.0	23.0	20.3	0.05	0.08	0.04	0.06	0.04	0.05
**TVC_p_**	NA	NA	NA	NA	NA	NA	15.6	14.2	16.8	19.2	15.0	18.9	0.06	0.07	0.06	0.05	0.07	0.05
**TEC**	NA	NA	26.1	20.0	24.7	NA	51.8	63.7	44.9	40.6	46.2	46.8	0.02	0.02	0.02	0.02	0.02	0.02
***Pseudomonas* spp.**	NA	NA	NA	NA	NA	NA	15.0	15.2	14.4	18.7	15.2	15.8	0.07	0.07	0.07	0.05	0.07	0.06
**LAB**	15.6	NA	59.3	25.3	103.7	68.6	27.8	30.9	26.2	21.9	36.6	14.6	0.04	0.03	0.04	0.05	0.03	0.07
***Br. thermosphacta***	NA	NA	NA	NA	82.9	1.82	16.6	17.0	17.8	18.5	96.6	14.9	0.06	0.06	0.06	0.05	0.07	0.07
***Photobacterium* spp.**	NA	NA	25.0	NA	28.2	47.5	17.8	17.4	18.7	15.9	16.2	17.3	0.06	0.06	0.05	0.06	0.06	0.06
**HSPB**	NA	NA	NA	NA	NA	NA	15.1	12.0	18.8	25.9	16.1	12.7	0.07	0.08	0.05	0.04	0.06	0.08

^1^ Calculated using the formula *G* = *t*/3.3 log*b*/*B*, where *t* = time interval in h to when the late lag phase was reached, *b* = number of bacteria at the end of the time interval, and *B* = number of bacteria at the beginning of the time interval [27]. ^2^ CTL = untreated control. ^3^ NA = not applicable as the bacteria were in the log phase of growth at time *t* = 0. The mean bacterial (TVC_m_, TVC_p_, TEC, *Pseudomonas* spp., LAB, *Br. thermosphacta*, *Photobacterium* spp. and HSPB) counts on cod treated with 1% (*v*/*v*) CIT, 1% (*v*/*v*) CAR, 1% (*w*/*v*) THY and 1% (*v*/*v*) EUG and stored at 2 °C are shown in Table 5. The corresponding growth parameters (initial lag period, mean generation time and maximum growth rate) are shown in Table 6. TVC_m_ and TVC_p_ counts were statistically similar (*P* > 0.05) to the control at each sampling time, regardless of treatment. Similarly, the other bacterial counts (TEC, *Pseudomonas* spp., LAB, *Br. thermosphacta*, *Photobacterium* spp. and HSPB) were not significantly different (*P* > 0.05) to the control within the bacterial groups with the following exceptions; *Br. thermosphacta* with CAR (*t* = 6) and HSPB with CAR (*t* = 3, 6, 9, 12, 15 and 18).

**Table 5:** Mean mesophilic total viable count (TVC_m_), psychrophilic total viable count (TVC_p_), total *Enterobacteriaceae* (TEC), *Pseudomonas* spp., lactic acid bacteria (LAB), *Br. thermosphacta*, *Photobacterium* spp. and hydrogen sulphide producing bacteria (HSPB) counts (log_10_ CFU/cm^2^) on cod treated with sterile distilled water (SDW), 1% (*v*/*v*) citral (CIT), 1% (*v*/*v*) carvacrol (CAR), 1% (*w*/*v*) thymol (THY) or 1% (*v*/*v*) eugenol (EUG) and stored at 2 °C for 18 days.

Time (days)	Treatment											
	CTL ^1^		SDW		CIT		CAR		THY		EUG	
	**TVC_m_**											
	Log	SE	Log	SE	Log	SE	Log	SE	Log	SE	Log	SE
**0**	3.1	0.4	2.8	0.3	3.1	0.2	2.4	0.1	3.2	0.1	3.0	0.2
**3**	3.9	0.5	3.9	0.3	3.9	0.4	3.3	0.5	3.5	0.9	3.7	0.7
**6**	5.2	0.6	5.5	0.4	5.8	0.3	5.3	0.3	5.5	0.4	5.6	0.3
**9**	6.4	0.5	6.6	0.4	6.9	0.4	6.9	0.5	6.8	0.4	6.9	0.4
**12**	7.1	0.3	7.2	0.3	7.6	0.2	7.4	0.2	7.5	0.3	7.5	0.2
**15**	7.9	0.1	7.8	0.2	8.1	0.1	8.0	0.1	7.9	0.2	8.1	0.2
**18**	7.8	0.3	7.8	0.3	8.1	0.3	8.3	0.4	8.0	0.2	8.4	0.4
	**TVC_p_**											
	Log	SE	Log	SE	Log	SE	Log	SE	Log	SE	Log	SE
**0**	3.5	0.2	3.0	0.2	3.3	0.1	2.6	0.2	3.1	0.2	3.2	0.1
**3**	4.3	0.3	4.2	0.2	4.2	0.1	3.7	0.5	4.3	0.4	4.3	0.3
**6**	5.7	0.3	5.8	0.2	6.2	0.1	5.8	0.3	6.2	0.3	6.2	0.1
**9**	7.1	0.1	7.0	0.2	7.4	0.1	7.2	0.2	7.3	0.2	7.4	0.1
**12**	7.8	0.1	7.9	0.2	8.3	0.1	8.0	0.2	7.9	0.2	8.2	0.0
**15**	8.2	0.0	8.3	0.3	8.2	0.1	8.7	0.4	8.3	0.1	8.3	0.1
**18**	8.4	0.2	8.4	0.2	8.4	0.2	8.4	0.2	8.4	0.2	8.6	0.2
	**TEC**											
	Log	SE	Log	SE	Log	SE	Log	SE	Log	SE	Log	SE
**0**	1.1	0.6	0.6	0.4	1.0	0.5	0.4	0.3	0.7	0.4	0.8	0.4
**3**	1.1	0.8	1.0	0.6	1.0	0.6	0.3	0.2	1.4	0.7	1.1	0.7
**6**	1.6	1.1	1.6	1.0	2.1	1.0	1.2	0.7	1.6	0.8	1.5	0.8
**9**	2.0	1.2	2.3	1.2	2.4	1.2	1.7	0.9	2.3	1.2	2.3	1.2
**12**	2.2	1.1	2.0	0.9	2.5	1.3	1.8	1.0	2.4	1.2	2.4	1.2
**15**	2.8	0.7	2.9	0.7	3.1	1.0	2.0	1.0	2.5	1.2	2.6	1.1
**18**	2.6	0.4	2.6	0.8	2.8	0.8	1.9	1.0	2.7	0.7	2.3	1.1
	***Pseudomonas* spp.**											
	Log	SE	Log	SE	Log	SE	Log	SE	Log	SE	Log	SE
**0**	2.9	0.2	2.3	0.2	2.8	0.2	1.9	0.3	2.2	0.3	2.3	0.2
**3**	4.1	0.2	4.3	0.2	4.5	0.1	3.9	0.4	4.5	0.3	4.4	0.3
**6**	6.0	0.4	6.2	0.3	6.5	0.0	6.0	0.3	6.2	0.2	6.4	0.1
**9**	7.3	0.2	7.2	0.2	7.5	0.1	7.3	0.2	7.3	0.2	7.3	0.1
**12**	7.8	0.2	7.9	0.3	8.5	0.3	7.9	0.3	8.1	0.0	8.1	0.0
**15**	8.2	0.1	8.4	0.3	8.4	0.1	8.6	0.3	8.2	0.1	8.2	0.2
**18**	8.7	0.1	8.8	0.1	8.6	0.0	8.6	0.1	8.3	0.1	8.8	0.1
	**LAB**											
	Log	SE	Log	SE	Log	SE	Log	SE	Log	SE	Log	SE
**0**	2.0	0.5	1.3	0.5	1.8	0.5	1.1	0.4	1.4	0.5	1.4	0.5
**3**	2.2	0.4	2.0	0.5	1.8	0.4	1.1	0.4	2.6	0.6	2.1	0.5
**6**	2.7	0.5	2.9	0.4	3.0	0.6	1.6	0.4	2.8	0.7	3.0	0.4
**9**	3.6	0.5	3.7	0.5	3.9	0.6	2.5	0.0	4.0	0.7	4.2	0.4
**12**	4.2	0.5	4.3	0.3	5.0	0.6	3.5	0.2	4.7	0.7	5.0	0.4
**15**	4.7	0.4	4.9	0.6	5.3	0.5	4.7	0.2	5.3	0.6	5.5	0.4
**18**	5.0	0.5	4.8	0.6	5.4	0.4	4.7	0.2	5.3	0.5	5.7	0.2
	***Br. thermosphacta***											
	Log	SE	Log	SE	Log	SE	Log	SE	Log	SE	Log	SE
**0**	1.6	0.1	0.9	0.5	1.6	0.2	0.7	0.4	1.3	0.3	0.9	0.1
**3**	2.8	0.2	2.8	0.4	2.8	0.1	2.0	0.2	3.2	0.5	3.0	0.3
**6**	4.4 ^B,2^	0.3	4.6 ^B^	0.3	4.3 ^B^	0.4	2.8 ^A^	0.2	4.4 ^B^	0.5	4.6 ^B^	0.1
**9**	5.7	0.5	5.9	0.4	5.7	0.4	4.7	0.6	5.6	0.5	5.8	0.3
**12**	6.1	0.4	6.6	0.3	6.5	0.3	5.5	0.1	6.5	0.4	6.6	0.2
**15**	7.2	0.4	7.3	0.4	6.9	0.3	6.4	0.0	6.9	0.4	6.8	0.5
**18**	7.1	0.2	7.4	0.2	7.0	0.2	6.4	0.1	6.8	0.3	7.0	0.1
	***Photobacterium* spp.**											
	Log	SE	Log	SE	Log	SE	Log	SE	Log	SE	Log	SE
**0**	3.5	0.2	3.0	0.3	3.3	0.1	2.5	0.2	3.0	0.1	3.0	0.2
**3**	4.0	0.3	3.9	0.1	3.9	0.1	3.1	0.4	4.3	0.3	4.2	0.3
**6**	5.2	0.6	5.5	0.3	6.1	0.0	5.2	0.5	5.7	0.3	6.2	0.3
**9**	6.6	0.3	6.9	0.3	7.4	0.1	7.3	0.2	7.3	0.3	7.4	0.1
**12**	7.6	0.2	7.9	0.2	8.4	0.3	8.1	0.3	8.0	0.1	8.1	0.0
**15**	8.2	0.1	8.4	0.1	8.3	0.3	8.4	0.3	8.3	0.1	8.3	0.1
**18**	8.6	0.1	8.7	0.2	8.4	0.1	8.4	0.2	8.5	0.1	8.6	0.1
	**HSPB**											
	Log	SE	Log	SE	Log	SE	Log	SE	Log	SE	Log	SE
**0**	2.4	0.3	1.6	0.5	1.8	0.2	1.0	0.2	1.6	0.1	1.4	0.2
**3**	4.8 ^B^	0.3	4.6 ^B^	0.4	3.7 ^B^	0.3	1.5 ^A^	0.2	4.0 ^B^	0.3	3.4 ^B^	0.3
**6**	5.5 ^B^	0.0	5.6 ^B^	0.2	5.6 ^B^	0.4	2.4 ^A^	0.8	5.5 ^B^	0.3	5.3 ^B^	0.4
**9**	7.1 ^B^	0.1	7.0 ^B^	0.2	6.9 ^B^	0.4	4.6 ^A^	0.7	6.6 ^B^	0.3	6.0 ^B^	0.6
**12**	7.3 ^B^	0.1	7.3 ^B^	0.3	7.2 ^B^	0.3	3.6 ^A^	1.3	7.4 ^B^	0.2	7.1 ^B^	0.1
**15**	7.5 ^B^	0.2	7.5 ^B^	0.2	7.1 ^B^	0.3	4.4 ^A^	0.8	7.3 ^B^	0.3	6.9 ^B^	0.1
**18**	7.5 ^B^	0.3	7.4 ^B^	0.3	6.8 ^B^	0.3	4.4 ^A^	0.5	7.1 ^B^	0.3	6.7 ^B^	0.2

^1^ CTL = untreated control. ^2^ Statistical analysis: At a given sampling time the same letter indicates no significant (*P* > 0.05) difference. Absence of a letter also indicates no significant (*P* > 0.05) difference.

**Table 6:** Growth parameters (initial lag period, mean generation time and maximum growth rate (µmax)) of mesophilic total viable count (TVC_m_), psychrophilic total viable count (TVC_p_), total *Enterobacteriaceae* (TEC), *Pseudomonas* spp., lactic acid bacteria (LAB), *Br. thermosphacta*, *Photobacterium* spp. and hydrogen sulphide producing bacteria (HSPB) on cod treated with sterile distilled water (SDW), 1% (*v*/*v*) citral (CIT), 1% (*v*/*v*) carvacrol (CAR), 1% (*w*/*v*) thymol (THY) or 1% (*v*/*v*) eugenol (EUG) and stored aerobically at 2 °C for 18 days.

Bacteria	Initial Lag Time (h)						Mean Generation Time (h) ^1^						µmax (Generations h^−1^)					
	CTL ^2^	SDW	CIT	CAR	THY	EUG	CTL	SDW	CIT	CAR	THY	EUG	CTL	SDW	CIT	CAR	THY	EUG
**TVC_m_**	24.1	NA ^3^	21.2	29.9	50.8	24.5	18.3	17.8	14.7	12.7	13.2	15.1	0.05	0.06	0.07	0.08	0.08	0.07
**TVC_p_**	29.2	NA	27.6	21.8	7.0	14.4	16.3	16.3	13.0	12.5	14.5	13.7	0.06	0.06	0.08	0.08	0.07	0.07
**TEC**	96.6	18.7	68.6	87.7	NA	101.1	39.0	39.4	25.8	24.4	42.4	20.7	0.03	0.03	0.04	0.04	0.02	0.05
***Pseudomonas* spp.**	NA	NA	NA	NA	NA	NA	15.2	17.0	14.5	13.3	15.0	14.4	0.07	0.06	0.07	0.08	0.07	0.07
**LAB**	80.9	18.1	73.0	127.3	NA	29.0	26.3	25.4	18.4	18.8	27.5	20.6	0.04	0.04	0.05	0.05	0.04	0.05
***Br. thermosphacta***	NA	NA	3.2	11.5	NA	NA	17.9	16.2	15.8	16.8	18.0	15.6	0.06	0.06	0.06	0.06	0.06	0.06
***Photobacterium* spp.**	42.2	18.9	47.4	55.1	9.2	11.8	17.3	15.5	11.6	10.1	14.7	13.6	0.06	0.06	0.09	0.10	0.07	0.07
**HSPB**	NA	NA	NA	78.0	NA	NA	19.4	17.4	11.4	12.7	15.8	15.4	0.05	0.06	0.09	0.08	0.06	0.07

^1^ Calculated using the formula *G* = *t*/3.3 log*b*/*B*, where *t* = time interval in h to when the late lag phase was reached, *b* = number of bacteria at the end of the time interval, and *B* = number of bacteria at the beginning of the time interval [27]. ^2^ CTL = untreated control. ^3^ NA = not applicable as the bacteria were in the log phase of growth at time *t* = 0.
